# Promoting Long-Term Parent and Caregiver Mental Health Through Universal Postnatal Nurse Home Visiting: Intervention Effects and Mechanisms of Action

**DOI:** 10.1007/s11121-025-01827-6

**Published:** 2025-08-01

**Authors:** Gayane A. Baziyants, Kenneth A. Dodge, W. Benjamin Goodman, Yu Bai, Robert A. Murphy, Karen O’Donnell

**Affiliations:** 1 Sanford School of Public Policy, Duke University, 201 Science Drive, Duke Box 90239, Durham, NC 27708 USA; 2 Department of Psychiatry and Behavioral Sciences, Duke University Medical Center, Box 102508, Durham, NC 27710 USA

**Keywords:** Home visiting, Parent mental health, Randomized control trial

## Abstract

Poor mental health affects millions of parents and caregivers each year. In the absence of intervention, the duration and magnitude of mental health symptoms can have an adverse impact on parent and caregiver well-being, parenting practices, and subsequent children’s development. Although home visiting is hypothesized to impact parent mental health, most studies do not demonstrate sustained benefits over time. Family Connects (FC) is a short-term, universal postnatal nurse home-visiting program designed to support children and families. Evaluations of FC demonstrate 6-month impacts on parent mental health, but longer-term (5-years post intervention) benefits have not been investigated, nor the potential mechanisms of the sustained effect. Every resident birthing family in Durham, NC, over an 18-month period (total *n* = 4777) was randomly assigned by birth date to FC or control condition. Implementation was strong, allowing an intent-to-treat evaluation of the model on maternal mental health. At infant age 60 months, a random, representative sample of parents (FC *n* = 201; control *n* = 200) was interviewed by condition-blind researchers with two screening instruments, the Center for Epidemiological Studies Depression Scale (CES-D) and the Mental Health Continuum scale (MHC – SF). Regression analyses with relevant covariates tested hypothesized (one-tailed) effects on these self-report scales. Parents randomly assigned to FC were significantly (*p* < .02) less likely to receive a depression score in the clinical range, reported a lower number of depressive symptoms (*p* < .04), and received better scores for social well-being (*p* < .04). Quality of the home environment (*p* < 0.10) was a significant mediator of intervention impact on later parent mental health.

**Supplementary Information:**

The online version contains supplementary material available at 10.1007/s11121-025-01827-6.

## Introduction and Background

Symptoms associated with depression affect over 7.5 million parents and caregivers every year (National Research Council (U.S.), ( 2009)). The daily context of navigating parenting demands, work conditions, and providing for basic needs of a household (e.g., food, clothing, shelter) can take a toll on any parenting adult (Lyons-Ruth et al., 2002; Smith & Mazure, 2021). As prior research suggests that 94% of families report at least one unmet psychosocial or financial need shortly after birth, early identification of caregiver needs may ensure timely provision of treatment or services that may reduce the duration of psychological symptoms (Dodge, 2022; National Institutes of Health, 2020). This manuscript reports findings from a randomized controlled trial of Family Connects (FC), a universal home visiting program, examining its effects on parent and caregiver (hereafter referred to as “parents”) mental health at infant age 5 years and the mechanisms underlying sustained outcomes.

### Child Development Consequences

Parenting that is responsive and attuned to a child’s cues and behaviors promotes the greatest likelihood of optimal development, while neglectful or indifferent parenting can be one of the most harmful (Hong & Park, 2012). In the presence of depression, parenting practices often become negative (e.g., hostile) and disengaged (e.g., withdrawal), limiting a parent’s ability to meet children’s growing needs (Goodman & Gotlib, 1999; Pelaez et al., 2008). Children living with a parent experiencing depression experience adverse impacts on health and development (Downey & Coyne, 1990). During infancy and early childhood, children are more likely to experience insecure attachment and decreased emotional regulation (Granat et al., 2017).

The negative outcomes of living with a parent suffering from depressive symptoms have been documented in meta-analyses indicating increases in children’s internalizing and externalizing behaviors (Goodman, 2007; Goodman et al., 2011). Children face a higher risk of developing psychiatric disorders or face challenges in their physical, cognitive, or emotional development (Beardselee et al., 1998). The physical environment and safety of the child can also become jeopardized as parents make fewer child doctor wellness visits, become less responsive to cues and basic needs, or withdraw from caregiving duties (Steadman et al., 2007).

### Causes of Poor Parent Mental Health

In the USA today, poor mental health is the leading cause of death among pregnant women and new mothers (Trost et al., 2024). Parent depression can be caused by a wide variety of factors, including genetic or biological dispositions as well as stressors caused by family or community contexts (Lewis et al., 2024). In the context of the family environment, parents may feel they do not have the skills to raise their children, leading to low levels of self-efficacy and parenting competence (Angley et al., 2015; Kohlhoff & Barnett, 2013). Parent inability to engage in positive parenting practices may also lead to increased symptoms of distress (Cornish et al., 2006; Crnic & Low, 2002). Parents who do not feel supported by their social or community networks are also at a higher risk for experiencing depressive symptoms due to lack of emotional, informational, or physical support (e.g., child care, time, financial assistance) (Corrigan et al., 2015). Demographic risk factors have also been documented, including being a new or single parent, living in low-income conditions, or belonging to a minority racial or ethnic group (Howell et al., 2005; Wszołek et al., 2018). Interventions and policy efforts aimed at supporting parents may promote improvements in mental health outcomes.

### Home Visiting and Parent Mental Health

Home visiting has been utilized as a preventive program for over a century (Astuto & Allen, 2009). Although different in their intended population and specific services provided, home visiting programs are motivated by the overall need to support families and promote positive children’s development (Administration for Children and Families, 2021). Home visiting can bypass possible limitations related to accessibility of services by directly providing services in the comfort of the individual’s home. In regard to parent mental health, home visiting has been hypothesized to improve parent mental health by providing an increase in supportive relationships, knowledge building, confidence, and self-efficacy (Ammerman et al., 2010). While some studies demonstrate positive, short-term impact of home visiting intervention on reduced depression, other studies indicate no support for this hypothesis (Ammerman et al., 2010; Duggan et al., 2007). Across numerous evidence-based home visiting programs in the U.S., parent mental health is the most challenging area to address (Duggan et al., 2018). Challenges noted by home visitors include parent mistrust, parent denial, limited home visitor training, and lack of program or community support availability (Duggan et al., 2018; Tandon et al., 2005).

### Family Connects

FC is a short-term, universal, postnatal nurse home-visiting program designed to support all parents in a community (Dodge et al., 2013). Originally launched in Durham, NC, FC has been scaled across 22 states since dissemination began in 2017 (Family Connects International, 2025). In contrast with other U.S. home visiting programs, FC is unique in both its universal offer and design. Upon giving birth, all parents in a defined catchment area (e.g., all resident births in a county or at a birthing hospital), regardless of race/ethnicity, income, or other demographic characteristics, are approached with the offer to participate in the home visiting program. If the parent agrees, a trained nurse home visitor visits the family in the comfort of their home. During the initial home visit, the nurse co-assesses with the family each of 12 categories across four domains of need, including material and financial, childcare, medical, and parenting needs (Dodge et al., 2014). If the nurse and family co-identify a type of need, the nurse either provides the intervention immediately (e.g., explains or demonstrates a needed concept, such as positioning for optimal latching during breastfeeding) or initiates a connection to a local community agency. After completing the initial home visit, families can receive 1–2 additional follow-up visits based on need and a phone call 4 weeks after case closure to assess family satisfaction and referral outcomes. Research on program implementation indicates 80% of all families agreed to participate and 86% of those families completed the program, with strong (90%) adherence by nurse home visitors to FC protocol (Goodman et al., 2021).

Prior results from the randomized control trial (RCT) demonstrate impacts across several domains. Compared with control families, families randomly assigned to FC demonstrate better parent mental health at child age 6 months (Dodge et al., 2014) and 2 years (Baziyants et al., 2023), and 39% fewer child maltreatment investigations through child age 5 years (Goodman et al., 2021). Economic analyses show that FC is a cost-beneficial approach for communities, with $3.17 in average savings for every $1 in program cost by 2 years of age (Goodman et al., 2019).

### Current Study

The present study examines the impact of FC on measures of parent mental health, 5 years post intervention. While prior research on HV programs has documented challenges in addressing mental health in parents due to factors of parent mistrust, limited provider training, and community support availability, implementation findings from FC have demonstrated positive receptivity by parents and provide compelling evidence of strong parent trust and high-fidelity service delivery. Over 98% of families reported discussions with the nurse as helpful and 95% found the nurse teachings to be beneficial (Dodge, et al., 2014). Importantly, over 99% of families stated they would recommend the program to others—underscoring high levels of trust. Fidelity data demonstrate that nurse home visitors are well-trained, with high adherence to protocol and strong interrater reliability in assessing family needs and risk levels. FC also demonstrates success in connecting families to services, with more than half of families successfully engaging with service providers following a referral, and 39% already receiving support for needed service by the time of the follow-up HV. Findings provide a strong rationale for the hypothesis that supporting all parents with a high-quality program shortly after childbirth can promote sustained effects on parent mental health.

The second research question is to identify any heterogeneity of FC impact across the different demographic characteristics of the families in the sample. The hypothesized finding is that single parents, minority status parents, or parents currently on Medicaid may experience a significant positive effect on mental health. The rationale for the hypothesis is that FC may promote reductions in parent stress and an increase in community support, which have been noted to be common challenges among these parents (Howell et al., 2005; McLearn et al., 2006).

A third research question is to identify mediators for the sustained effect (if a main effect is observed). Hypothesized mechanisms of impacts on parent mental health through home visiting have pointed to factors of social support, knowledge building, confidence, and self-efficacy in parenting domains (Ammerman et al., 2010). Given FC impact on parenting, home environment quality, and mental health outcomes at child age 6 months and 24 months, mediation analyses will test whether these factors account for the sustained effect (Baziyants et al., 2023; Corrigan et al., 2015; Dodge et al., 2014; Respler-Herman et al., 2012).

## Methods

### Study Design

FC was evaluated through a RCT consisting of all 4777 resident families of infants born between July 1, 2009, and December 31, 2010, in Durham County, North Carolina. Families were assigned to treatment and control conditions a priori based on infant birth date. If the child was born on an even date, the family was invited to participate in the FC program. If the child was born on an odd date, the family received community “services as usual.” A representative subsample was randomly selected using public birth records for evaluation of long-term impact findings at child age 6 months (Dodge et al., 2014). To remove any bias that may jeopardize study goals, evaluation-study families were informed this was a study of infant development. If the family declined to participate, another child was randomly selected with the same birth date and race/ethnicity. The final subsample consisted of 549 families, representing one birth for each day of the 18-month RCT implementation period. Of these families, 18 were subsequently dropped because of errors in hospital records regarding date of birth, yielding a final sample of 531 families, as outlined in the CONSORT diagram by Dodge and authors (2019). Families were contacted for follow-up interviews at child age 60 months (+/– 2 months). This 5-year in-home interview addressed a wide variety of domains, including maternal physical and mental health, parenting, parent–child relationships, and children’s health and development. For the present study, data were analyzed only for the parent mental health domains. This study is registered with ClinicalTrials.gov, NCT01406184, https://clinicaltrials.gov/ct2/show/NCT01406184, and the Duke School of Medicine Institutional Review Board (IRB) approved all study procedures.

### Participants

Participants at child age 5 years included 401 families (76% of the 531 initially assigned). Of this sample, 201 families were in the treated group (75% retention) and 200 families were in the control group (71% retention). There were no statistically significant differences in retention between the treated and control families (Table S1). Families not retained for the final study sample were more likely than those retained to be on Medicaid or uninsured or of minority status (Table S1). Hospital discharge data were utilized to gather demographic characteristics of the families in the study. Of the 401 families in the study, 12.5% had a recorded birth risk, 39.2% were of single-parent households, 68.6% were of minority status, and 60.6% received Medicaid or were uninsured (Table S1). Additionally, 98.3% were biological parents, 1.2% were grandparents, 0.25% were adoptive parents, and 0.25% were other. Parent interviews reflect one respondent, with 97.76% retained from the original 6-month interview.

### Measures

Parent mental health was measured using the Center for Epidemiological Studies Depression Scale (CES-D) and the Mental Health Continuum Scale (MHC – SF) (Keyes et al., 2008; Radloff, 1977). Measures are described in detail in the Supplementary Materials.

#### CES-D

##### Parent Depressive Symptoms

The mean of 20 items reflecting depressive symptoms was scored with a range from 0 (rarely or none of the time (less than 1 day in the past week)) to 3 (most or all of the time (5–7 days in the past week), with higher scores reflecting greater depressive symptoms (alpha = 0.91). Scores were standardized.

##### Possible Clinical Depression

A dichotomous score reflecting possible clinical depression (0 = not depressed; 1 = possible depression) was computed based on previously established guidelines using a sum score of 16 or higher (Lewinsohn et al., 1997).

#### MHC—SF

##### Overall Mental Health

The mean of 14 items reflecting overall mental health was computed based on responses. Scores ranged from 0 (never in the past month) to 5 (every day in the past month), with higher scores reflecting greater overall mental health (alpha = 0.94).

##### Happiness

The mean of 3 items reflecting happiness was computed based on responses. Scores ranged from 0 (never in the past month) to 5 (every day in the past month), with higher scores reflecting greater happiness (alpha = 0.87).

##### Social Well-Being

The mean of 5 items reflecting social well-being was computed based on responses. Scores ranged from 0 (never in the past month) to 5 (every day in the past month), with higher scores reflecting greater social well-being (alpha = 0.84).

##### Psychological Well-Being

The mean of 6 items reflecting psychological well-being was computed based on responses. Scores ranged from 0 (never in the past month) to 5 (every day in the past month), with higher scores reflecting greater psychological well-being (alpha = 0.91).

### Demographic Variables

Demographic variables were collected from hospital discharge data for all children. These variables served as covariates in the main effect analysis. Demographic variables included *parents*’ *single parent status* (0 = no; 1 = yes), *parents*’ *health insurance status* (0 = private insurance; 1 = Medicaid/no insurance), *minority status* (0 = nonminority race/ethnicity; 1 = minority race/ethnicity), *infant medical risk at birth* (comprised of sum scores of infant birth weight, infant gestational age, or other medical risks as defined by the International Classification of Diseases), *and infant gender* (0 = boy; 1 = girl). The selected demographic variables have been recognized as risk factors for parental depression and included in other FC studies (Baziyants et al., 2023; Dodge et al., 2019; Howell et al., 2005; Wszołek et al., 2018).

### Mediator Variables

Parent outcomes at child age 6 months and child age 24 months were utilized to test for mediation. Positive parenting was measured by the Warmth and Parenting Behaviors Questionnaire, which included age-appropriate questionnaire items identified from the Mother–Child Neglect Scale (Lounds et al., 2004), the Parent–Child Conflict Tactics Scale (Straus et al., 1998), and the Durham Family Initiative Cross-Site Interview (Durham Family Initiative, 2008). At child age 6 months, home environment was observed by condition-blinded interviewers who utilized the Duke Endowment Child Abuse Prevention Initiative Neighborhood Survey (Daro & Dworsky, 2005). Parent mental health was measured through the Edinburgh Postnatal Depression Scale (Cox et al., 1987). Example questions are listed in the Supplementary Materials.

#### Positive Parenting (Child Age 6 Months)

A mean of 7 questionnaire items reflecting self-reported measures. Scores ranged from 0 (never in the past month) to 5 (> 20 times in the past month), with higher scores reflecting greater positive parenting behaviors.

#### Home Environment Quality (Child Age 6 Months)

A mean of 5 questionnaire items reflecting observer ratings. Scores ranged from 1 (inadequate) to 7 (excellent), with higher scores reflecting greater quality of the home environment.

#### Possible Clinical Depression (Child Age 6 Months)

A dichotomous score reflecting possible clinical depression (0 = not depressed; 1 = possible depression) was computed based on a sum score greater than 10, based on previously established guidelines (Cox et al., 1987).

#### Positive Parenting (Child Age 24 Months)

A mean of 7 questionnaire items reflecting self-reported measures. Scores ranged from 0 (never in the past month) to 5 (> 20 times in the past month), with higher scores reflecting greater positive parenting behaviors.

### Statistical Analysis

The main effect of random assignment to FC was analyzed using Ordinary Least Squares (OLS) regression models. The OLS model equation is as follows:

$${Y}_{i}={\beta }_{0}+ {\beta }_{1}Treate{d}_{i}+ \sum_{m=2}^{m=5}{\beta }_{m }{X}_{mi}+ {\epsilon }_{i}$$

where ${Y}_{i}$ represents the average score for the parent mental health outcome variables, ${\beta }_{0}$ is a constant, ${\beta }_{1}$ is the average effect of random assignment to FC (0 = no, 1 = yes), ${Treated}_{i}$ is a dummy indicator for assignment to FC, ${X}_{i}$ are covariates, and ${\epsilon }_{i}$ is the error term. The main effect analysis included five demographic covariates. Prior longitudinal studies of FC have demonstrated improvements in parent mental health, leading to the formulation of a directional hypothesis for the present study (Baziyants et al., 2023; Dodge et al., 2019). Based on these prior empirical findings, a one-tailed test at the *p* < 0.05 level was declared to be significant.

Moderation analyses were conducted to explore possible heterogeneity of the main effects of FC across the demographic characteristics of parents in the sample, which also informed outcomes for potential subgroup analyses. Models were motivated by previously published FC studies that found a significant interaction between random assignment and family demographics on outcomes of child maltreatment, emergency care visit, positive parenting, and parenting sense of competence (Baziyants et al., 2023; Dodge et al., 2013; Goodman et al., 2019, 2021). Consistent with other FC evaluations, moderation analyses were estimated for each of the five demographic categories. Given the exploratory nature of the moderation analyses, with no a priori hypothesis, a two-tailed test at *p* < 0.05 was declared to be significant and *p* < 0.10 was declared to be marginally significant. To reduce the risk of Type I error as a result of 30 moderation tests, a Holm–Bonferroni sequential correction was applied, with a post hoc test of significance applied following Aiken and West (1991; Holm, 1979). Subgroup analyses were conducted, with a two-tailed test at *p* < 0.05 declared significant for outcomes that were at least marginally significant from moderation analyses. Missing values were identified for one variable, Medicaid/uninsured status (15 cases). Consequently, multiple imputation was implemented to create 10 data sets to handle missing data (Schafer & Graham, 2002).

To evaluate potential mechanisms of sustained effects of FC on outcomes of parent mental health, mediational analyses were conducted utilizing previously published parent outcomes from child age 6 months and child age 24 months (Baziyants et al., 2023; Dodge et al., 2014). The final set of mediators included parent-reported positive parenting at child age 6 months and 24 months, as well as observer-rated quality of the home environment at child age 6 months (Table S3). Three single mediator tests were conducted with a latent outcome variable that was generated to represent parent mental health at 60 months. The latent variable included the three main effect outcomes that were significant or marginally significant from the main effect findings from the first research question (Table 1). Model fit was tested with root mean square error of approximation (RMSEA) less than 0.001 and comparative fit index (CFI) 1.000, which confirmed the fit of the latent variable construct (Hu & Bentler, (Hu, and Bentler, 1999)). A bootstrap method (1000 bootstrap samples) was utilized to conduct mediation analyses, which has been recommended for obtaining robust estimates of the indirect effect and confidence intervals (MacKinnon et al., (MacKinnon et al., 2004); Preacher & Hayes, (Preacher, and Hayes, 2008); Zhang et al., (Zhang et al., 2015)). To handle missing data, MI was utilized alongside the bootstrap approach, as described in Zhang and authors (2015).

**Table 1:** Main effects of random assignment to treatment

Outcome	Main effect				
	*b (s.e.)*	One-sided 95% CI	Two-tail *p* value	One-tail *p* value	Cohen’s *d*
Parents depression	− 0.17** (0.10)	(- Inf, − 0.01)	0.07	0.04	0.20
Possible clinical depression^a^	− 0.52** (0.25)	(Inf, 0.90)	0.04	0.02	0.59^b^
Overall mental health	0.16* (0.10)	(**− **0.01, Inf)	0.12	0.06	** − **0.17
Happiness score	0.15* (0.10)	(**− **0.02, Inf)	0.15	0.07	** − **0.17
Social well-being score	0.18** (0.10)	(0.02, Inf)	0.07	0.04	** − **0.20
Psychological well-being	0.10 (0.10)	(**− **0.06, Inf)	0.31	0.15	** − **0.11

*N* = 401

All variables presented in Table 1 are controlled for in the model

Robust standard errors in parenthesis

Multiple imputation (*m* = 10) was used to handle missing data

Cohen’s *d* = (mean of control group mean of treatment group)/pooled standard deviation)

*CI* confidence interval, *Inf* infinity.

CI reflect one-sided 95% bounds for one-tailed tests

^a^Model estimated using logistic regression

^b^Odds ratio is reported

***p < 0.01; **p < 0.05; *p < 0.1

## Results

### Main Effects

#### Parent Depressive Symptoms

Random assignment to FC was associated with a significantly lower dimensional score for self-reported parent depressive symptoms (B =  − 0.17; 95% CI $:$—Inf, − 0.01; *p* < 0.05, *d* = 0.20, Table 1). Moderation analyses did not indicate a significant interaction of treatment status and parent demographic characteristics.

#### Possible Clinical Depression

Parents randomly assigned to FC were significantly less likely to be diagnosed with possible clinical depression when compared to control parents (odds ratio = 0.59; 95% CI: Inf, 0.90; *p* < 0.05, Table 1). Moderation analyses did not indicate a significant interaction of treatment status and parent demographic characteristics.

#### Overall Mental Health

Random assignment to FC was associated with a marginally significant higher score for overall mental health (B = 0.16; 95% CI: − 0.01, Inf; *p* < 0.10, d =  − 0.17, Table 1). Moderation analyses did not indicate a significant interaction of treatment status and parent demographic characteristics.

#### Happiness

Random assignment to FC was associated with a marginally significant higher score for happiness (B = 0.15; 95% CI: − 0.02, Inf; *p* < 0.10, *d* =  − 0.17, Table 1). Moderation analyses did not indicate a significant interaction of treatment status and demographic characteristics.

#### Social Well-Being

Random assignment to FC was associated with significantly higher scores for social well-being (B = 0.18; 95% CI: 0.02, Inf; *p* < 0.05, d =  − 0.20, Table 1). Moderation analyses indicated a significant interaction between treatment status and infant gender (B = 0.55; 95% CI = 0.20, 0.90; *p* < 0.05). Subgroup analyses demonstrate a significant positive effect for parents with a female child (B = 0.44; 95% CI = 0.20, 0.70; *p* < 0.01).

#### Psychological Well-Being

There were no significant differences in parents who were randomly assigned to FC relative to parents who were not in scores for psychological well-being. Moderation analyses did not indicate a significant interaction of treatment status and parent demographic characteristics.

#### Mediational Findings

Measures of positive parenting at child age 6 months and 24 months did not significantly mediate the effect of random assignment to FC on parent mental health at child age 5 years. Home quality environment at child age 6 months may be a potential mechanism through which positive impacts on parent mental health are sustained by 11% (Table 2).

**Table 2:** Direct and indirect effects of Family Connects

Mediator	Indirect effect	95% Bootstrap confidence interval	Percentage of total impact accounted by mediator	Direct effect	Total effect	Observations
6 mo						
Positive parenting	0.01 (0.01)	− 0.00, 0.02	11.11%	0.08 (0.02)***	0.09 (0.03)***	401
Home environment	0.01 (0.00)*	− 0.00, 0.01	11.11%	0.08 (0.02)***	0.09 (0.02)***	401
24 mo						
Positive parenting	0.00 (0.01)	− 0.01, 0.01	0%	0.08 (0.05)	0.08 (0.05)	395

Independent variable = treatment status; Dependent variable: latent construct of child age 60 months parent depression, possible clinical depression, and social well-being

Three single mediator models were tested, each model included the same covariates as the main effect analyses

Bootstrapped replications of 1000; Standard error in parenthesis

*p < 0.1; **p < 0.05; ***p < 0.01

## Discussion

Study findings demonstrate that parents randomly assigned to FC reported significantly fewer depressive symptoms and were less likely to receive a depression score in the clinical range relative to parents who were not randomly assigned to FC. Parents randomly assigned to FC were also more likely to report greater social well-being relative to parents in the control group. These findings are encouraging in the context of both policy and HV program interventions, as many HV programs demonstrate only short-term impacts on mental health, with relatively few demonstrating sustained impacts of up to 5 years (Ammerman et al., 2010; Miles et al., 2025). Nonetheless, there was not a significant effect of FC on the measures of parents’ overall mental health and happiness. The contrast between FC impact on outcomes of depression and social well-being relative to other mental health outcomes may indicate the important role FC has in supporting parents in their transition to parenthood. Depression and social isolation can be common symptoms experienced by parents in their transition to parenthood (Bauman et al., 2020). By assessing families across different categories of need and providing support or services, FC may be shortening the duration and severity of depression and social isolation. Other mental health outcomes like happiness, overall mental health, and psychological well-being, are reflective of broader aspects of parents’ lives (e.g., self-acceptance), which may be more challenging to sustain long-term with the early intervention services provided by FC.

Mediational analyses demonstrate that FC positive impacts on the quality of the home environment at child age 6 months may partially explain the sustained improvements in parent mental health observed 5 years post-intervention. The home environment measure captured indicators of household safety, cleanliness, and the availability of age-appropriate toys and learning materials, which is highly contingent on parent knowledge of child health and safety, which is an important aspect of parenting competency (Gadsden et al., 2016). Parents with acquired knowledge on how to meet children’s basic needs and prevent injuries are more likely to create a safe, nurturing environment, which can mitigate the development of emotional and financial stressors. Such stressors may stem from preventable incidents that would result in unintended emergency room visits or maltreatment investigations, which have been linked to poor mental health symptoms in parents (Kuang et al., 2018). These findings may imply that timely provision of support related to the home environment—such as improvements in parent knowledge, confidence, and material resources (e.g., books)—may be a mechanism through which FC contributed to the sustained improvements in parent mental health over time.

The observed effect sizes across outcomes of parent depressive symptoms, possible clinical depression, and social well-being, while small, have important preventative implications. The clinical relevance of an intervention should be measured by a ratio of the intervention effect size divided by the cost of the intervention (Cuijpers et al., 2014; Huhn et al., 2014). The cost of the FC intervention was about $500 per family in the population. In the context of mental health, even small improvements can mitigate the risk, duration, severity, and cost of more serious mental health symptoms, promoting positive effects for parents and children (National Institutes of Health, 2020). Moreover, small improvements applied at the population level can have positive implications for public health more broadly, especially when effects are sustained over time. FC is a light-touch program, consisting of 1 to 3 home visits, between child age 3 weeks through 12 weeks (Dodge et al., 2014). Cost–benefit analyses focused solely on reductions in infant emergency care visits have demonstrated the potential of community savings exceeding six million dollars in just the first 6 months of the infant’s life (Dodge et al., 2014). Future work should consider replicating cost–benefit analyses with outcomes of parent mental health.

Subgroup analyses suggest positive effects hold across all subgroups studied, including single and two-parent families, health-insured and non-insured families, minoritized and non-minoritized families, families whose infant was at medical risk at birth and those not at medical risk, and infant males and females. Only one exception was found for one variable: Assignment to intervention had a positive impact on social well-being among parents with a female child but not among those with a male child. These findings are surprising given limited empirical evidence of a differential effect of home visiting programs based on child gender. Broader evidence from early childhood programs suggests that girls at baseline have lower family resources and less favorable home conditions, demonstrating why girls may benefit more from interventions than boys (García et al., 2018). Alternatively, the gender differences may also be due to differences in parenting or disciplinary practices for male and female children. For example, research shows that parents tend to engage in more strict parenting with boys relative to girls (McKee et al., 2007). The differences in discipline may end up increasing parent stress, inadvertently reducing any positive effect of the intervention on parent mental health outcomes.

## Limitations

There are several limitations of this study. First, all measures of parent mental health were assessed using self-reported scores by the parents. Given the self-reported nature of data collection, responses may be subject to social desirability bias and recall bias (Paulhus, 1984; Raphael, 1987; Widom et al., 2004). Future studies should consider adding observational measures or nurse administered screenings to the parent mental health assessment. Second, the study sample is limited to that of Durham, North Carolina families. Although Durham is comprised of diverse families (White 54.7%, Black 35.3%, Hispanic or Latino 13.9%), future research should consider similar longitudinal evaluations with larger sample sizes that are reflective of other states of the country (U.S. Census Bureau, 2023). Third, the study sample focuses solely on one parent, limiting the ability to differentiate between mental health outcomes in two-headed households. During the FC pilot study, while both parents were actively invited to participate, challenges related to inter-parent conflicts and scheduling difficulties led several families to be hesitant to participate in the program. Consequently, program and research efforts were adjusted to prioritize mothers as the primary parent or caregiver (Dodge & Goodman, 2019). Given evidence of pronounced gender disparities in mental health, future research should examine the well-being of both parents to understand effective methods for supporting both parents in a household (Metzger & Gracia, 2023). Additionally, one-tailed tests have been noted to be employed incorrectly in empirical studies, and future studies should consider whether the use of one-tailed tests is appropriate in similar program evaluations (Ringwalt et al., 2011). For this study, the rationale for the use of one-tailed (unregistered) tests was justified based on prior empirical evidence pointing to a positive effect of FC on parent mental health. Lastly, no analyses were conducted to assess spillover effects. While the study design was randomized, it did not fully account for the possibility that parents in the same network (e.g., other family members, neighbors) may have learned about the universal home visiting program and subsequently benefited from it. This could have reduced the observed significant main effect in the study.

## Conclusion

The findings from this study demonstrate a sustained 5-year impact of a universal, short-term, nurse home visiting program on parent depression and well-being. The positive impact of FC on improving the quality of the home environment at child age 6 months partially mediates the sustained effects on parent mental health. The sustained 5-year impact on parent mental health has important implications on children’s development and demonstrates a cost-effective approach to promoting long-term parent mental health.

## Supplementary Information

Below is the link to the electronic supplementary material.

Supplementary file1 (DOCX 45 KB)

## Data Availability

De-identified data files can be made available to qualified researchers upon request.
