# Supplementary material for: Promoting Long-Term Parent and Caregiver Mental Health Through Universal Postnatal Nurse Home Visiting: Intervention Effects and Mechanisms of Action
Source: Prev Sci. 2025 Aug 1;26(6):921–31. doi: 10.1007/s11121-025-01827-6 (PMC12345448; doi:10.1007/s11121-025-01827-6)
Supplement: Supplementary file 1 — Supplementary file1 (DOCX 45 KB) [file 11121_2025_1827_MOESM1_ESM.docx]

**Supplementary Materials**

Measures

The CES-D and MHC-SF have been validated as reliable measures of parent mental health in various populations and contexts (Beekman et al., 1997; Ferentinos et al., 2019; Franken et al., 2018; Hann et al., 1999; Jiang et al., 2019; Lim, 2022; Radloff, 1977). Although each measure provides an indication of mental health, the domains and utility vary. The CES-D assesses primarily depressed affect, positive affect, somatic symptoms, and interpersonal difficulties (Radloff, 1977). MHC-SF focuses on emotional well-being, psychological well-being, and social well-being (Keyes et al., 2008). Utilizing both measures in assessing parent mental health can provide a more comprehensive evaluation of mental health conditions, which is important for individualizing intervention service delivery.

***CES-D***

**Parent Depressive Symptoms.** Example questions included “I felt depressed”, “I felt sad”, and “I was bothered by things that usually don’t bother me”.

**Possible Clinical Depression.** A dichotomous score reflecting possible clinical depression (0: not depressed; 1 = possible depression) was computed based on a sum score of 16 or higher, based on previously established guidelines (Lewinsohn et al., 1997).

***MHC - SF***

**Overall Mental Health.** Example questions asked how often in the past month the parent felt “happy”, “satisfied with life”, and “confident to think or express your own ideas and opinions”.

**Happiness.** Example questions asked how often in the past month the parent felt “happy”, “interested in life”, and “satisfied with life”.

**Social Well-Being.** Example questions asked how often in the past month the parent felt that “you had something important to contribute to society”, “you belonged to a community (like a social group or neighborhood)”, and “our society is a good place, or is becoming a better place, for all people”.

**Psychological Well-Being.** Example questions asked how often in the past month the parent felt “that you liked most parts of your personality”, “good at managing the responsibilities of your daily life”, and “confident to think or express your own ideas and opinions”.

***Mediator Variables***

**Positive Parenting (child age 6 months)**. Example questions included, “Read books to [CHILD]”, “Comforted [CHILD] when he or she was upset”, “Explained why something she/he did was wrong”.

**Home Environment Quality (child age 6 months).** Example questions included, “Overall, the home is safe, clean, and free of hazards”, “Overall, basic hygiene standards are maintained”, “Was there a variety of books, age-appropriate toys or learning materials?”.

**Positive Parenting (child age 24 months).** Example questions included, “Helped [CHILD] when he/she was upset”, “Praised [CHILD]”, “Did things just for fun with [CHILD] (went to a fun place like a playground)”.

Tables and Figures

Table S1. Baseline characteristics of families

|  | **Treatment and Control Families** | | | | | **Retained and Attrited Families** | | | | |
| --- | --- | --- | --- | --- | --- | --- | --- | --- | --- | --- |
| **Characteristics** | **Control** | | **Treatment** | |  | **Retained** | | **Dropped** | |  |
|  | **n** | **Percent** | **n** | **Percent** | **pvalue** | **n** | **Percent** | **n** | **Percent** | **pvalue** |
| Birth risk ever |  |  |  |  |  |  |  |  |  |  |
| No | 171 | 85.50 | 180 | 89.55 | 0.219 | 351 | 87.53 | 129 | 86.58 | 0.745 |
| Yes | 29 | 14.50 | 21 | 10.45 | . | 50 | 12.47 | 20 | 13.42 | . |
| Total | 200 | 100.00 | 201 | 100.00 | . | 401 | 100.00 | 149 | 100.00 | . |
| Infant female |  |  |  |  |  |  |  |  |  |  |
| No | 88 | 44.00 | 101 | 50.25 | 0.210 | 189 | 47.13 | 71 | 47.65 | 0.861 |
| Yes | 112 | 56.00 | 100 | 49.75 | . | 212 | 52.87 | 78 | 52.35 | . |
| Total | 200 | 100.00 | 201 | 100.00 | . | 401 | 100.00 | 149 | 100.00 | . |
| Single parent |  |  |  |  |  |  |  |  |  |  |
| No | 118 | 59.00 | 126 | 62.69 | 0.450 | 244 | 60.85 | 88 | 59.46 | 0.768 |
| Yes | 82 | 41.00 | 75 | 37.31 | . | 157 | 39.15 | 60 | 40.54 | . |
| Total | 200 | 100.00 | 201 | 100.00 | . | 401 | 100.00 | 148 | 100.00 | . |
| Medicaid/uninsured |  |  |  |  |  |  |  |  |  |  |
| No | 75 | 38.46 | 77 | 40.31 | 0.710 | 152 | 39.38 | 30 | 20.55 | <0.001 |
| Yes | 120 | 61.54 | 114 | 59.69 | . | 234 | 60.62 | 116 | 79.45 | . |
| Total | 195 | 100.00 | 191 | 100.00 | . | 386 | 100.00 | 146 | 100.00 | . |
| Minority status |  |  |  |  |  |  |  |  |  |  |
| No | 58 | 29.00 | 68 | 33.83 | 0.297 | 126 | 31.42 | 24 | 16.11 | <0.001 |
| Yes | 142 | 71.00 | 133 | 66.17 | . | 275 | 68.58 | 125 | 83.89 | . |
| Total | 200 | 100.00 | 201 | 100.00 | . | 401 | 100.00 | 149 | 100.00 | . |

Notes:

The P value comes from X2 test between the control and treatment group.

* p<0.1, ** p<0.05, *** p<0.01

Table S2. Treatment and control group descriptive statistics across outcomes

| **Outcome** | **Control** | |  | **Treatment** | |
| --- | --- | --- | --- | --- | --- |
|  | **Mean ± SD or %** | **N** |  | **Mean ± SD or %** | **N** |
| Parent Depressive Symptoms | 0.53 ± 0.49 | 200 |  | 0.43 ± 0.45 | 201 |
| Possible Clinical Depression | 25.00 | 200 |  | 16.42 | 201 |
| Overall Mental Health | 3.80 ± 0.93 | 200 |  | 3.96 ± 0.86 | 201 |
| Happiness Score | 4.11 ± 0.84 | 200 |  | 4.25 ± 0.85 | 201 |
| Social Well-Being Score | 3.19 ± 1.26 | 200 |  | 3.43 ± 1.15 | 201 |
| Psychological Well-Being | 4.15 ± 0.97 | 200 |  | 4.25 ± 0.87 | 201 |

Table S3. Means of potential mediator measures across treatment and control groups

| **Mediator Variables** | **Control** | **Treatment** | **Combined Means** | **Total** | **Test** |
| --- | --- | --- | --- | --- | --- |
| **Child age 6 months** |  |  |  |  |  |
| Positive Parenting | 4.05 (0.42) | 4.12 (0.44) | 4.08 (0.43) | 401 | 0.10 |
| Home Environment Quality | 4.60 (1.50) | 4.89 (1.50) | 4.74 (1.51) | 377 | 0.06 |
| Home Environment Matrix | 2.61 (1.18) | 2.55 (1.16) | 2.58 (1.17) | 400 | 0.58 |
| EPDS Possible Depression Dichotomous Score^a^ | 61.36 | 38.64 | 10.97 | 401 | 0.11 |
| EPDS Mean Score | 0.44 (0.46) | 0.40 (0.44) | 0.42 (0.45) | 401 | 0.39 |
| **Child age 24 months** |  |  |  |  |  |
| Positive Parenting | 4.51 (0.47) | 4.60 (0.46) | 4.55 (0.47) | 395 | 0.05 |

Notes:

The P-value comes from two-sample X2 test between the treatment and control group for categorical variables and t-tests for continuous variables

^a^ Percent is reported for dichotomous variable

EDPS: Edinburgh Postnatal Depression Scale

Standard deviation in parenthesis

*** p<0.01, ** p<0.05, * p<0.1
